# High‐dose‐rate brachytherapy for prostate cancer: Rationale, current applications, and clinical outcome

**DOI:** 10.1002/cnr2.1450

**Published:** 2021-06-23

**Authors:** Iosif Strouthos, Efstratios Karagiannis, Nikolaos Zamboglou, Konstantinos Ferentinos

**Affiliations:** ^1^ Department of Radiation Oncology German Oncology Center Limassol Cyprus; ^2^ Clinical Faculty, School of Medicine European University Cyprus Nicosia Cyprus

**Keywords:** combined with EBRT, high‐dose‐rate, interstitial brachytherapy, monotherapy, prostate cancer, salvage

## Abstract

**Background:**

High‐dose‐rate brachytherapy (HDR BRT) has been enjoying rapid acceptance as a treatment modality offered to selected prostate cancer patients devoid of risk group, employed either in monotherapy setting or combined with external beam radiation therapy (EBRT) and is currently one of the most active clinical research areas.

**Recent findings:**

This review encompasses all the current evidence to support the use of HDR BRT in various clinical scenario and shines light to the HDR BRT rationale, as an ultimately conformal dose delivery method enabling safe dose escalation to the prostate.

**Conclusion:**

Valid long‐term data, both in regard to the oncologic outcomes and toxicity profile, support the current clinical indication spectrum of HDR BRT. At the same time, this serves as solid, rigid ground for emerging therapeutic applications, allowing the technique to remain in the spotlight alongside stereotactic radiosurgery.

## 1 INTRODUCTION

Validated therapeutic modalities considered for patients diagnosed with organ‐confined prostate cancer are radical prostatectomy,^1^, ^2^ external beam radiation therapy (EBRT),^3^, ^4^, ^5^ permanent low‐dose rate (LDR) brachytherapy (BRT),^6^, ^7^, ^8^ and temporary high‐dose‐rate (HDR) BRT.^9^, ^10^, ^11^, ^12^, ^13^, ^14^, ^15^, ^16^, ^17^, ^18^, ^19^ However, owing to the unavailability of randomized clinical trials, the optimal management remains trivial, with proposed treatment assignment being mainly determined by physician's biased guidance and patient's preference. In this regard, choice of treatment and consecutively its impact on quality of life have gained increasing importance, with BRT being favored due to its high effectiveness and, at the same time, its relatively low morbidity. Currently, validated long‐term data endorse the efficacy of BRT in the management of locally confined prostate cancer with technological advancements fueling active research in the field of HDR BRT owed mainly to refinement of the technique,^20^ employment of modern biomolecular imaging,^21^, ^22^, ^23^ and investigation of focal and focused approaches,^24^ all of which ensure high standards of implant quality and precision. The dosimetric superiority of HDR BRT translates into excellent clinical results,^25^, ^26^, ^27^ thus backing up the notion that HDR BRT is a novel alternative to permanent LDR BRT.^28^

This review presents a comprehensive analysis of the rationale, current clinical indications, and oncologic outcomes, including a representative data report.

## 2 BACKGROUND

### 2.1 Rationale for HDR brachytherapy

Dose escalation data suggest that the utilization of comparatively higher dose for definitive radiation therapy (RT) in organ‐confined prostate adenocarcinoma improves biochemical control (BC)^4^, ^5^, ^29^ but, at the same time, results in improved metastasis‐free survival (MFS).^5^, ^30^, ^31^, ^32^, ^33^, ^34^ Adding to that, the rational assumption can be made that further therapeutic impact improvement could be attained through dose escalation, while simultaneously enhancing dose conformity, especially in patients devoid of regionally advanced and/or metastatic tumor load. HDR BRT fully exploits its radiobiological advantage to perfectly meet this objective, through the utilization of extreme hypofractionation^35^, ^36^, ^37^ and, at the same time, its incomparably superior three‐dimensional (3D) dosimetry.^38^ HDR treatment planning enables dose optimization through multiparametric modulation, for example, catheter geometry, precalculated dwell positions, and times.^39^, ^40^ This allows for optimal dose modulation, with higher dose delivery to target volume and/or selectively dose reduction to organs at risk (OARs).^25^

In relation, HDR BRT employs “high‐density” dosimetry, owed to the roughly twofold dwell positions number when compared to seeds in a typical LDR implant. Again in comparison to LDR, anatomic and, thus, dosimetric changes are kept to a minimum, since issues associated with LDR BRT such as migration of seed/source and deformation of tissue do not occur.^41^, ^42^, ^43^

On the other hand, intrafractional anatomic alteration caused by organ motion during EBRT delivery,^44^, ^45^, ^46^ as well as setup inaccuracies, is overcomed with HDR due to rectification of theses error during the implantation procedure with interactive online dosimetry or modified prior to dose delivery with real‐time anatomy‐based treatment planning.^25^

This minimization of errors allows for a decrease in the therapeutic margins required beyond the intended target, thus exposing less healthy tissue in unnecessary radiation, transforming HDR BRT to the optimal intraprostatic dose‐escalation technique, where needed, especially when combined with EBRT. This proved especially important in patients whose treatment volume includes the regional lymphatic drainage, being treated to a moderate dose, yet offering an escalated intraprostatic escalated dose.

### 2.2 Radiobiological considerations

Radiobiological data suggest that there is variability between normal and malignant tissue and the probability of acute and late radiation sequelae development, variation which is also being noted in‐between different fractionation schedules. Adhering to the linear‐quadratic model,^47^ the sensitivity of a particular tissue to altered fraction size is expressed by the α/β ratio, allowing comparison between various treatment schedules and, at the same time, estimates the impact of each given fractionation schedule on tumor control and toxicity. Recent radiobiological reports suggest an α/β ratio for prostate cancer ranging between 1.2 and 3.0 Gy, which is relatively lower than the α/β ratio of acutely and late‐reacting normal tissues.^36^, ^48^, ^49^ Having this in mind, hypofractionated dose schemes are favored and seem to result in superior tumor control with remarkable reduction in late side effects. In this background, HDR BRT represents the ideal method for conformal dose escalation.^50^

### 2.3 Patient selection for HDR brachytherapy

Based on the hypothesis that failure of local control in organ‐confined prostate cancer may lead to regional and distant metastasis development, histologically confirmed localized disease is the fundamental indication for HDR BRT in patients, who are considered suitable candidates for definite treatment.^51^, ^52^

In line with the National Comprehensive Cancer Network (NCCN) guidelines,^53^ patients with low‐ and intermediate‐risk are stratified as optimal candidates for local radical treatment, considering they bear the highest probability for organ‐confined prostatic disease. Concomitantly, reports from mature retrospective series encourage the use of HDR BRT monotherapy in a selection of high‐risk patients, based on the notion that the therapeutic margin provided is superior to RP, with OARs' dose (urinary bladder and rectum) remaining significantly lower in comparison with definitive dose‐escalated EBRT plans.

On the other hand, in patients stratified as intermediate and high risk,^1^, ^53^, ^54^ the utilization of combined HDR BRT as a boost modality with EBRT is a well‐established treatment supported by valid data.^55^, ^56^, ^57^, ^58^ Again, HDR BRT may find implementation in the regional lymphadenopathy setting, with or without the presence of distant metastatic spread, as a combination with EBRT as part of an individualized treatment concept, aiming at minimizing toxicity, with the goal of maximizing local disease control.

In the local recurrence setting after definitive RT, as proposed by international guidelines,^51^, ^52^, ^59^ any patient presenting histological and/or radiological (also biomolecular imaging) proved prostate‐confined disease is a potential candidate for local radical treatment, therefore prostate salvage HDR BRT (sHDR BRT) should be considered.

Prior to HDR BRT, complete clinical staging should be attempted following the European Association of Urology,^60^ European Society for Radiotherapy and Oncology (ESTRO),^52^ and American Brachytherapy Society (ABS)^51^ guidelines. Patient's precise group stratification and further on choice of therapeutic modality should be based on thorough clinical work‐up, consisting of histological confirmation of the prostatic malignancy, and clinical investigations for evaluation of possible disease spread, including digital rectal examination, transrectal ultrasound (TRUS), computed tomography (CT), bone scintigraphy, and/or magnetic resonance imaging (MRI). In uncertain cases of regional lymphadenopathy, laparoscopic pelvic lymphadenectomy or positron emission tomography may be considered for optimal staging.

Although the baseline urinary function can be predictive for functional outcome following HDR BRT,^61^ neither larger glandular size nor previous transurethral resection of the prostate (TURP) (given a sufficient amount of time has surpassed [>3 months] and residual gland volume remains for image‐based 3D treatment planning),^62^, ^63^, ^64^ should be considered as absolute contraindications.

When comparing HDR to LDR or EBRT, the exacerbation of lower urinary tract symptoms appears to be less prolonged, based on the fact that even patients with high International Prostate Symptom Score (IPSS; ≥20) tend to have a rather rapid recovery to pretreatment baseline urinary function.^65^ Selection criteria for HDR BRT as monotherapy, combined with EBRT and in the salvage setting, are presented in Table 1.

**TABLE 1:** Patient selection criteria for HDR BRT in the treatment of prostate cancer

Inclusion criteria
Stages cT1‐T3b^a^
Any Gleason score
Any PSA level
Exclusion criteria
TURP within 3 months
IPSS >20
Pubic arch interference
Lithotomy position not possible^b^
Anaesthesia not possible
Rectal fistula

Abbreviations: IPSS, International Prostate Symptom Score; TURP, transurethral resection of the prostate.

^a^ Selected T4 tumors included with curative intent in the protocols of selected centers.

^b^ Relevant for TRUS‐guided technique, does not apply for MRI‐guided implantation.

In contrary to permanent LDR implants, HDR BRT after loading catheters can be implanted accordingly, in order to cover areas of extracapsular or the seminal vesicles' infiltration or even the bladder pouch, extending its indication to coverage of even T4 tumors, as part of individualized curative treatment concepts.^14^, ^66^, ^67^ Previous pelvic EBRT, prior pelvic surgery, and inflammatory bowel disease are not considered absolute contraindications for HDR prostate BRT but always a very thorough evaluation of the potential risks and benefits should take place, based on anatomy‐based dosimetry including carefully defined OARs dose constraints.^25^

### 2.4 Implantation techniques

Anaesthesia, spinal or general, is required for interstitial catheter implantation. It should be stated that catheter implantation can be carried out using TRUS‐guided technique,^13^, ^68^, ^69^ where extensive experience exists or by MRI‐assistance.^52^, ^53^ Table 2 describes key features of the technique.

**TABLE 2:** Key features in the HDR BRT of prostate cancer

Important steps for high‐dose‐rate brachytherapy are:	
A.	Catheter placement under image guidance (usually TRUS)
B.	Imaging with catheters in place: TRUS, CT, or MRI
C.	Definition/contouring of CTV, OARs, and catheter reconstruction on planning system Current step might include image co‐registration aiding at gross disease delineation: TRUS, MRI, PET
D.	Dwell position and time optimisation
E.	Quality assurance tests
F.	Treatment delivery

Abbreviations: CT = computed tomography; CTV = clinical target volume; MRI = magnetic resonance imaging; OARs = organs‐at‐risk; PET = positron emission tomography; TRUS = transrectal ultrasound.

**FIGURE 1:**
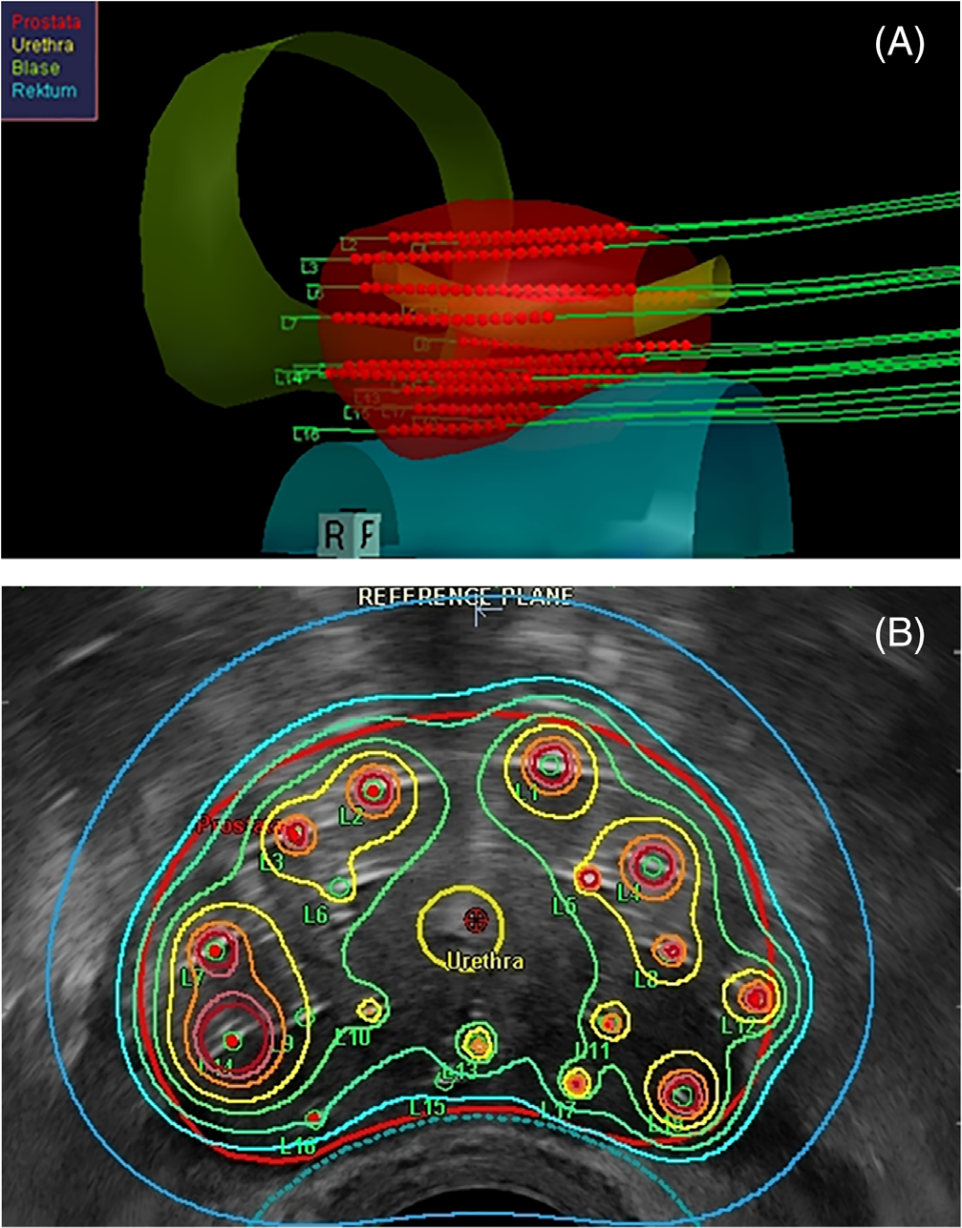
(A) Three‐dimensional reconstruction of the prostate, urethra, rectum, and bladder with template catheter trajectories for TRUS‐guided implantation as calculated for preplanning by the real‐time treatment planning system SWIFT/Oncentra Prostate (Nucletron – an Elekta Company, B.V., Veenendaal, The Netherlands). (B) Intraoperative real‐time TRUS‐based treatment planning presenting isodose distribution after anatomy‐based dose optimization. The isodose color code convention is dark red = 300 %, orange = 200 %, yellow = 150 %, green = 125 %, turquoise = 100 %, and dark blue = 50 %

In the TRUS‐based technique, implantation is carried out transperineally with the patient placed in high lithotomy position, using a template to aid catheter placement and a continuous probe movement technique. The clinical workflow includes image acquisition of the prostate, urethra, and anterior rectal wall and the creation of virtual volumes prior to implantation for inverse treatment preplanning.^40^ Three‐dimensional (3D) volume reconstruction follows based on a 0.1 cm image distance. Contouring commences based on the GEC/ESTRO guidelines.^52^ Abiding on the acquired 3D anatomy, precalculated virtual catheter positions are generated, activating catheter source dwell positions located within the PTV, while radioactive source dwell times are calculated using an intraoperative treatment planning system (Figure 1). Using a dose–volume histogram (DVH) of the PTV and the OARs (ie, intraprostatic urethra, anterior rectal wall, and urinary bladder), the final evaluation of the anatomy‐oriented dose optimization^39^ is performed. Once the dosimetric protocol parameters are met, TRUS‐guided implantation is carried out at the predefined catheter positions (Figure 1).

**FIGURE 2:**
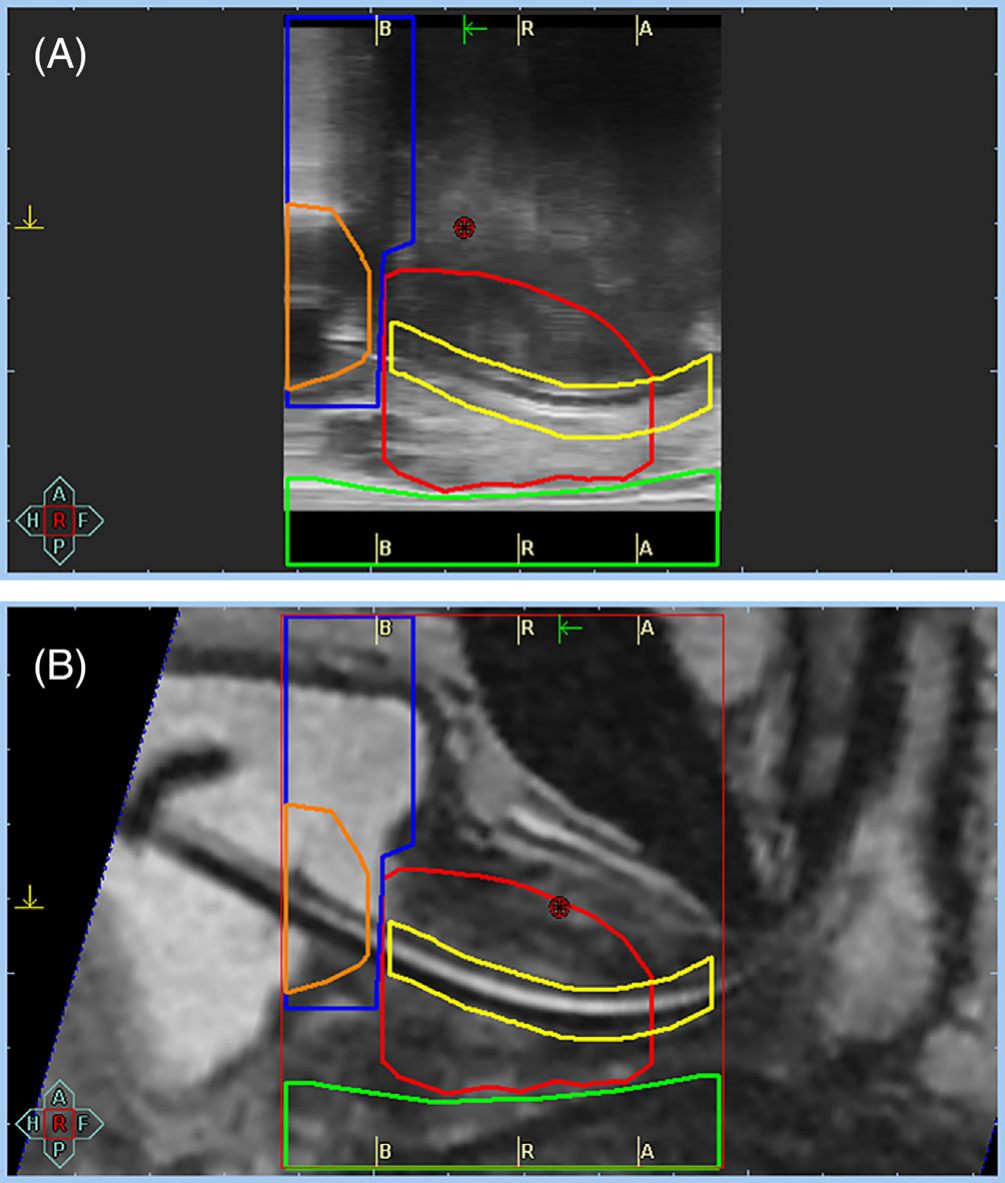
Image fusion with (A) ultrasound (US) image acquisition prior to interstitial catheter implantation and (B) magnetic resonance imaging (MRI) acquired on day of brachytherapy session with urethral catheter in place. Urethral catheter's balloon serves as a mark indicating the vesicourethral junction, a point easily identifiable both on US‐ and MRI‐images. MRI images assist in visibility of the prostatic base and apex. *blue contour = urinary bladder, red = prostate, yellow = urethra, green = rectum, orange = catheter balloon

In the MRI‐based implantation procedure, transperineal catheter placement is achieved by placing the patient in left lateral decubitus position, again employing a template device. The MRI‐based procedure parallels the workflow of TRUS‐guided implantation, since it involves a preplanning step based on 3D image reconstruction from the acquired preinterventional MRI sequences (of at least 0.3 cm slice thickness). The number, distribution, as well as distance between the catheters are predetermined by the preplanning which calculates the peripheral catheter arrangement with arbitrary optimization for target coverage. The maximum insertion depth and positional verification of the implanted catheters is performed by interactive MRI scanning following catheter implantation. An attempt to obtain the optimum from both worlds has already been made. In our department, a T2‐MRI sequence, with a placed urinary catheter, is obtained just before the TRUS‐guided transperineal implantation procedure begins. Based on clearly visible landmarks, such as the urinary catheter balloon, the vesicourethral junction can be easily identified, both on MRI and US images, aiding in optimal fusion of the two modalities and thereby precise prostate capsule definition, especially of the prostatic apex and base (Figure 2).

## 3 CLINICAL DATA

### 3.1 HDR brachytherapy in combination with EBRT

Dose‐escalation trials, in reference to the management of intermediate‐ and high‐risk prostate cancer, identified a marked improvement, observed both in BC as well as MFS.^4^, ^5^, ^29^, ^30^, ^32^, ^33^, ^34^, ^70^, ^71^, ^72^, ^73^ It is evident that the combination of EBRT with hypofractionated HDR BRT as a boost enables for safe delivery of high biologically equivalent doses to the prostate, which currently cannot be matched by any form of image‐guided EBRT.^29^, ^74^, ^75^, ^76^ Of particular importance is the comparison of HDR BRT with stereotactic approaches, in terms of conformality, proving its dosimetric superiority.^77^, ^78^

Randomized studies in confluence with mature retrospective data justify the superiority of combined modalities over EBRT alone in the primary treatment of localized high‐risk prostate adenocarcinoma. A randomized prospective study^55^ allocated 220 patients to either combined HDR BRT with hypofractionated EBRT or EBRT alone. The EBRT‐only scheme (*n* = 111) consisted of 55 Gy administered over 20 fractions, whereas in the combined group (*n* = 109) of 35.75 Gy, EBRT was administered over 13 fractions followed by a 17‐Gy HDR boost applied in two fractions with a single implant. The combined arm proved superior in regard to mean biochemical failure‐free survival, 5.1 years versus 4.3 years in the EBRT‐only group (*P* = .03), with higher‐grade GU as well as GI toxicity not reaching statistical significance. In an earlier study,^56^ 104 patient were randomized to either conventional EBRT up to a total physical dose of 66 Gy in 33 fractions or to 35 Gy pulse‐dose‐rate BRT delivered over 48 h plus EBRT of 40 Gy in 20 fractions 2 weeks later. A recent update of this study,^79^ with a median follow‐up of 14 years, reported an overall survival benefit for the combined technique, 67% in the EBRT arm compared to 77% in the combined modality arm, again without statistically significant differences in late GU and GI toxicity. Although BC remained improved in favor of the combined modality, unfortunately it did not manage to achieve statistical significance, owning mainly to the fact that the trial was underpowered. The recent ASCENDE RT Trial^80^ put two‐dose escalation methods to the test, with patients being allocated between a standard arm (*n* = 200) consisting of ADT for 12 months and pelvic EBRT to 46 Gy plus a EBRT boost to 78 Gy and an experimental arm (*n* = 198) employing an LDR BRT boost with minimal peripheral prostatic dose of 115 Gy. Achieving a median follow‐up of 6.5 years, the 7‐year biochemical failure‐free survival was in favor of the BRT arm, 86% compared to 75% in the EBRT arm. The favorable oncologic outcomes of the study were associated with higher rates of acute and late GU toxicity in the LDR boost‐arm, attaining a 5‐year cumulative incidence of grade 3 GU of 18.4% for LDR BRT vs 5.2% for the EBRT boost (*P* < .001).^57^

More recently, the TROG 03.04 RADAR study^81^ randomized patients with intermediate‐ (33%) and high‐risk (66%) disease to either 6 or 18 months of leuprorelin with or without 18 months of zoledronic acid. Patients were either treated solely by EBRT (66 Gy, 70 Gy, or 74 Gy) or received a high‐dose‐rate (HDR) brachytherapy boost (19.5 Gy in three fractions). In a multivariate analysis adjusted for age, use of zoledronic acid, and other validated prognostic stratification variables, the HDR boost subgroup reported that longer duration of androgen suppression (18 months) was associated with reduced distant progression, prostate‐cancer‐specific mortality, and all‐cause mortality (sHR, 0.61, 0.67, 0.59, respectively). Again, interestingly, the HDR boost was associated with reduced PCSM risk and improved overall survival, reaching statistical significance (*P* < .001 for both).

Adding to that, one of the largest retrospective series from our group^19^ included 303 high‐risk patients treated with EBRT delivering 45.0 Gy followed by an HDR BRT boost consisting of two fractions of 10.5‐Gy. The reported 7‐year biochemical relapse‐free survival and metastasis‐free survival rates were 88% and 93%, respectively. The reported incidence of late grade 3 GU adverse events was 2.2%, with no GI grade 3 being reported.

Acknowledging the methodological advantages of HDR BRT in comparison with LDR, in regard to the very steep fall‐off in dose beyond the PTV together with the versality of intratarget dose modulation, the avoidance of systematic errors, and imprecision in dose application due to anatomic deformities and source migration, it is only reasonable to state that all LDR outcomes can be reproducible, if not superior,^82^ with the employment of HDR BRT.

Overall, the heterogeneity of clinically implemented treatment schemes poses a great challenge, especially if attempting to propose uniform recommendations for a standardized protocol. That set aside, the published oncological results on combined RT are both consistent and reproducible (Table 3). The majority of institutions employ total physical HDR doses of 12–21 Gy applied in two to four fractions, with BRT fractions ranging from 6 to 10.5 Gy. The supplemental EBRT doses range from 45 to 54 Gy (normofractionation), generating total BED 1.5 and EQD2 doses in the range of 171–366 Gy and 74–137 Gy, respectively.^9^, ^10^, ^14^, ^15^, ^16^, ^56^, ^59^, ^68^, ^69^, ^79^, ^83^, ^84^, ^85^, ^86^, ^87^, ^88^, ^89^, ^90^, ^91^, ^92^, ^93^, ^94^, ^95^, ^96^, ^97^, ^98^, ^99^, ^100^, ^101^, ^102^, ^103^, ^104^, ^105^, ^106^, ^107^ The reported severe late GU and GI adverse events rates compare favorably with late toxicity rates in dose‐escalated EBRT series.^71^, ^105^, ^106^, ^108^ It must be noted that hypofractionated EBRT protocols are gaining momentum,^94^, ^95^ appearing equieffective in regard to clinical outcome, while demonstrating favorable toxicity profile.

**TABLE 3:** Literature results of HDR BRT as boost modality to EBRT for clinically localised prostate cancer

		Treatment scheme				
Study	*n*	Total EBRT dose (Gy/fx)	Total HDR dose (Gy/fx)	Total BED/EQD2 (Gy)	Follow‐up (y)	Biochemical control^a^
Galalae et al.^83^	122	40/20	18/2	219/94	Median 8.2	74% LR, 69% all‐risk groups at 5 years/8 years
Phan et al.^84^	309	46/23	36/18–50.4/28	191–218/82–94	Median 4.7	98% LR, 90% IR, 78% HR at 6 years
Pellizzon et al.^85^	209	36–54/20–30	16–24/4	138–239/59–102	Median 5.3	94.2% All‐risk groups at 3.3 years with 91.5% LR, 90.2% IR and 88.5% HR
Viani et al.^86^	131	45–50/25	16–24/4–6	158–205/68–88	Median 5.2	81% at 5 years with 87% IR and 71% HR
Morton et al.^87^	123	37.5/15	15/1	265/114	Median 1.2	100% All‐risk groups
Neviani et al.^88^	455	45/25	16.5–21/3	176–216/76–93	Median 4.0	92% LR, 88% IR, 85% HR at 4 years
Noda et al.^89^	59	50/25	15–18/2	207–243/89–104	Median 5.1	100% LR at 5 years, 92% IR at 5 years, 72% HR at 5 years
Agoston et al.^59^	100	40–60/20–30	8/1 or 10/1	144–217/62–93	Median 5.1	85.5% for all risk groups at 5 years with IR 84.2% at 7 years and HR 81.6% at 7 years
Martinez et al.^90^	472	46/23	16.5–23/2–3	184–306/79–131	Mean 8.2	56.9% All risk groups for EQD2 < 93 Gy and 81.1% all‐risk groups for EQD2 > 93 Gy at 10 years
Prada et al.^16^	313	46/23	23/2	306/131	Median 5.7	100% LR, 88% IR, 79%–81% HR
Aluwini et al.^91^	264	45/25	18/3	189/81	Median 6.2	97% LR and IR at 7 years
Whalley et al.^92^	101	46/23	17/2–19.5/3	211–220/91–95	Median 4.7	95% IR, 68% HR at 4 years
Kotecha et al.^14^	229	45/25–50.4/28	16.5–22.5/3	176–245/74–105	Median 5.1	95% LR at 7 years, 90% IR at 7 years, 57% HR at 7 years (81% HR with BED>190 Gy)
Hoskin et al.^13^	218	35.75/13	17/2	214/92	Median 7.1	75%, 66% and 46% for all‐risk groups at 5‐, 7‐ and 10‐years
Helou et al.^93^	123 60	37.5/15 45/25	15/1 20/2	265/114 252/108	Median 6.2 Median 8.5	97.4% at 5 years with IR 92.7% at 5 years with IR
Vigneault et al.^94^	832	40–44/20	18/3–15/1	184–274/79–117	Median 5.5	95% LR, 95% IR, 94% HR at 6 years
Ishiyama et al.^95^	3424	39/13	18/2	243/104	Median 5.5	91% IR, 81% HR at 10 years
Falk et al.^96^	159	46/23	18/3 or 18/2 or 14/1	223–177/100–76	Median 6.0	92% IR, 85% HR at 5 years
Strouthos et al.^19^	314	45/25	21/2	267/114	Median 5.9	86.3% for the entire cohort, 85.6% HR at 6 years

Abbreviations: BED, biologically effective dose considering an a/β‐ratio for prostate cancer of 1.5 Gy, EQD2, equieffective dose administered in 2Gy‐fractions; HR, high‐risk group; IR, intermediate‐risk group; LR, low‐risk group; y, years.

^a^ Biochemical failure defined by the *Phoenix definition* unless specified otherwise.

### 3.2 HDR monotherapy

As already mentioned, HDR BRT was originally used in combination with EBRT, as a boost modality mainly due to concerns regarding normal tissue toxicity with the application of hypofractionated treatment regimes. The safety and efficacy range for HDR in the context of combined EBRT and BRT have been clearly established by dose escalation trials.^106^, ^109^, ^111^ At the same time, the employment of other locally directed treatments such as RP, radical EBRT, and LDR BRT, together with the acknowledgment that image‐guided HDR with its anatomy‐based dose optimization offers high precision in prostate dose coverage, while simultaneously minimization of the total dose to adjacent OARs^25^ laid the way for broad practice of HDR BRT in the monotherapy setting. An evergrowing body of literature considers HDR safe and effective radical treatment with consistent intermediate‐ and long‐term BC rates over a range of risk groups.^13^, ^17^, ^18^, ^69^, ^112^, ^113^, ^114^, ^115^, ^116^, ^117^, ^118^, ^119^, ^120^, ^121^, ^122^, ^123^, ^124^, ^125^, ^126^ Although, moderate hypofraction enjoys the longest follow‐up in regard to clinical results (four to nine fractions), consistent data are reported for extreme hypofractionated protocols (one to three fractions). It should be noted that ultrahypofractionated attempts^115^, ^127^, ^128^ (one fraction) to make HDR logistically comparable with LDR BRT have proven inferior in respect to clinical outcomes and require further validation.^115^, ^129^, ^130^, ^131^, ^132^

Again, due to the variation of clinically implemented dose fractionation regimens, direct comparisons are proving difficult. Despite that, the oncological outcomes yielded for both single‐ and multiple‐implant schemes for extreme or moderated hypofractionated treatment protocols are uniform (Table 4).

**TABLE 4:** Oncological results of HDR monotherapy for localised prostate cancer

		HDR protocol						
Study	*n*	Gy/Fraction	Fractions (implants)	Total	median f/u (y)	Biochemical control^a^	BED (Gy)	EQD2 (Gy)
Morton et al.^114^	170	19 Gy 13.5 Gy	1 (1 implant) 2 (2 implants)	19 Gy 27 Gy	6.0	73.5% LR/IR at 5 years 95% LR/IR at 5 years	260–270	111–116
Strouthos et al.^18^	450	11.5 Gy	3 (3 Implant)	34.5 Gy	4.7	96% LR, 96% IR, 92% HR at 5 years	299	128
Hoskin et al.^128^	293	19–20 Gy 13 Gy 10.5 Gy	1 (1 implant) 2 (1 implant) 3 (1 implant)	19–20 Gy 26 Gy 31.5 Gy	4.1 5.3 9.0	94% IR/HR at 4 years 77% IR/HR at 7 years 81% IR/HR at 7 years	251–260	108–111
Krauss et al.^127^	58	19 Gy	1 (1 implant)	19 Gy	2.9	93% LR/IR at 3 years	260	111
Yoshioka et al.^114^	190	6.0 Gy 6.0 Gy 6.5 Gy	8 (1 Implant) 9 (1 Implant) 7 (1 Implant)	48 Gy 54 Gy 45.5 Gy	7.6	93% IR, 81% HR at 5 years	240–270	103–116
Hauswald et al.^113^	448	7.0–7.25 Gy	6 (2 Implants)	42–43.5 Gy	6.5	98.9% LR, 95.2% IR at 10 years	238–253	102–108
Jawad et al.^112^	494	9.5 Gy 12.0 Gy 13.5 Gy	4 (1 Implant) 2 (1–2 Implants) 2 (1–2 Implants)	38 Gy 24 Gy 27 Gy	4.1	98% LR, 95% IR at 5 years 92% LR, 81% IR at 5 years 100% LR,79% IR at 5 years	270–279	115–119
Prada et al.^115^	60	19.0 Gy	1 (1 Implant)	19 Gy	6.0	66% LR, 63% IR at 6 years	260	111
Kukiełka et al^116^	77	15.0 Gy	3 (3 Implants)	45 Gy	4.7	96.7% all risk groups at 5 years	495	212
Komiya et al.^121^	51	6.5 Gy	7 (1 Implant)	45.5	1.4	94% all risk groups at 17 months	243	104
Hoskin et al.^13^	197	8.5–9.0 Gy 10.5 Gy 13.0 Gy	4 (1 Implant) 3 (1 Implant) 2 (1 Implant)	34–36 Gy 31.5 Gy 26 Gy	3.1	95% IR, 87% HR at 4 years	227–252	97–108
Rogers et al.^119^	284	6.5 Gy	6 (2 Implants)	39 Gy	2.7	94% IR at 5 years	208	89
Zamboglou et al.^17^	718	9.5 Gy 9.5 Gy 11.5 Gy	4 (1 Implant) 4 (2 Implants) 3 (3 Implants)	38 Gy 38 Gy 34.5 Gy	4.4	95% LR, 93% IR 93% HR at 5 years	279–299	119–128
Barkati et al.^118^	79	10–11.5 Gy	3 (1 Implant)	30–34.5 Gy	3.3	85.1% LR/IR at 5 years	230–299	99–128
Demanes et al.^69^	298	7.0 Gy 9.5 Gy	6 (2 Implants) 4 (1 Implant)	42 Gy 38 Gy	5.2	97% LR/IR at 5 years	238–279	102–119
Mark et al.^117^	301	7.5 Gy	6 (2 Implants)	45 Gy	8.0	88% All risk groups at 8 years	270	117
Martinez et al^120^	248	7.0 Gy 9.5 Gy	6 (2 Implants) 4 (1 Implant)	42 Gy 38 Gy	4.8	87% LR/IR at 5 years 91% LR/IR at 5 years	238–279	102–119
Ghadjar et al.^88^	36	9.5 Gy	4 (1 Implant)	38 Gy	3.0	100% LR/IR at 3 years	279	119
Grills et al.^123^	65	9.5 Gy	4 (1 Implant)	38 Gy	2.9	98% LR/IR at 3 years	279	119

Abbreviations: BED, biologically effective dose considering an a/β‐ratio for prostate cancer of 1.5; EQD2, equieffective dose administered in 2.0 Gy‐fractions considering an a/β‐ratio for prostate cancer of 1.5 Gy; f/u, follow‐up; HR, high‐risk group; IR, intermediate‐risk group; LR, low‐risk group; y, years.

^a^ Biochemical failure defined by the *Phoenix definition*.

A great retrospective study^113^ focusing on 448 patients with low‐/intermediate‐risk disease treated with six fractions in two implants (spaced 1 week apart) to a median of 43.5 Gy. Temporary ADT was administered in 42 patients (9%). The actuarial 6‐ and 10‐year overall BC rate was 98.6% and 97.8%, respectively, with a median follow‐up of 6.5 years, while no significant difference in respect to biochemical progression‐free survival being noted at 10 years between low‐ and intermediate‐risk group (98.9% vs. 95.2%). Late grade 3–4 GU toxicity was 4.7%, with one patient (0.2%) experiencing grade 4 toxicity, while no late grade 3–4 GI toxicity was observed. These results are in line with experience from other major institutions suggesting that HDR BRT in the monotherapy setting can be applicable both for intermediate‐ and selected high‐risk disease cases.^13^, ^113^, ^114^, ^121^, ^123^, ^124^, ^125^ The Offenbach group^17^ in Germany reported on 718 consecutive patients, considered to this day, one of the largest patient collectives, administering three different protocols (four fractions of 9.5 Gy in single implant, four fractions of 9.5 Gy in two implants, and three fractions of 11.5 in three implants). Intermediate‐ and high‐risk patients made up 44.9% of the collective, with 60% of high‐ and 27% of intermediate‐risk cases receiving temporary ADT. The 5‐year BC rate was 93% and 93% for intermediate‐ and high‐risk patients, respectively. Late grade 3 GU and GI were reported at 3.5% and 1.6%, respectively.

Erectile dysfunction following BRT monotherapy has been rarely reported, using various multidimensional or ordinal scales for assessment. However, potency preservation rates of 60%–90% have been documented in recent literature.^17^, ^26^, ^113^, ^115^, ^119^, ^120^, ^121^, ^122^, ^123^, ^133^ In the previously mentioned series by Hauswald et al.,^113^ 315 (70%) patients managed to attain an erection sufficient for intercourse before treatment. A total of 225 patients provided data in regard to sexual function reaching a median of 6 years following treatment. An ability to engage in sexual intercourse, with or without the use of erectile aids, was reported by 60% of patients with median age of 69 years at time of assessment.

To date, only nonrandomized evaluations have put LDR and HDR monotherapy in comparison in regard to their toxicity profile and justified that high‐grade toxicities, both acute and late, are in favor of HDR.^120^, ^123^ A comparative, retrospective study^120^ analyzed and compared HDR monotherapy (*n* = 248) and LDR seed patients (*n* = 206), indicating that temporary HDR is being associated with significantly less grade 1–2 GU toxicity, in the form of chronic dysuria (LDR 22% vs. HDR 15%) and urinary frequency/urgency (LDR 54% vs. HDR 43%). The incidence of urethral stricture was equal for both therapeutic modalities (LDR 2.5% vs. HDR 3%), while late Grade 3 GU sequelae was insignificant in both groups. At last, the 5‐year potency preservation rate was 80% for temporary HDR versus 70% for permanent seeds BRT.

Overall, the reproducible clinical data in favor of HDR monotherapy clearly reflect the current radiobiological notion for optimal tumor control through hypofractionation. Table 5 describes sufficiently the biologically effective dose (BED) values, ranging from 208 to 299 Gy, with a median value of 256 Gy (*α*/*β‐*ratio of 1.5 Gy). Calculation of the EQD2 doses provides values of the range from 89 to 128 Gy, tendering such dose coverage unachievable with the current EBRT techniques.

**TABLE 5:** Late toxicity data of HDR monotherapy for localised prostate cancer

		HDR protocol			Toxicity			
Study	n	Gy/Fraction	Fractions (implants)	Total	GU Grade 2 (%)	GU Grade 3 (%)	GI Grade 2 (%)	GI Grade 3 (%)
Morton et al.^114^	170	19.0 Gy 13.5 Gy	1 (1 Implant) 2 (2 Implants)	19.0 Gy 27.0 Gy	45	1	1	0
Strouthos et al.^18^	450	11.5 Gy	3 (3 Implants)	34.5 Gy	14	0.8	0.4	0
Hoskin et al.^128^	293	19.0–20.0 Gy 13.0 Gy 10.5 Gy	1 (1 Implant) 2 (1 Implant) 3 (1 Implant)	19.0–20.0 Gy 26.0 Gy 31.5 Gy	2.6 0 2.1	2.6 0 1.1	0 3.5 0	0 0 0
Krauss et al.^127^	58	19.0 Gy	1 (1 Implant)	19.0 Gy	10.3	0	3.4	0
Yoshioka et al.^114^	190	6.0 Gy 6.0 Gy 6.5 Gy	8 (1 Implant) 9 (1 Implant) 7 (1 Implant)	48.0 Gy 54.0 Gy 45.5 Gy	6	2	4	2
Hauswald et al.^113^	448	7.0–7.25 Gy	6 (2 Implants)	42–43.5 Gy	–	4.7	–	0
Jawad et al.^112^	494	9.5 Gy 12.0 Gy 13.5 Gy	4 (1 Implant) 2 (1–2 Implants) 2 (1–2 Implants)	38.0 Gy 24.0 Gy 27.0 Gy	20	1	2	0
Prada et al.^115^	60	19.0 Gy	1 (1 Implant)	19.0 Gy	0	0	0	0
Kukiełka et al^116^	77	15.0 Gy	3 (3 Implants)	45.0 Gy	25	0	0	0
Komiya et al.^121^	51	6.5 Gy	7 (1 Implant)	45.5	QoL (IPSS, FACT‐P and IIEF) at baseline after 12 weeks			
Hoskin et al.^13^	197	8.5–9.0 Gy 10.5 Gy 13.0 Gy	4 (1 Implant) 3 (1 Implant) 2 (1 Implant)	34–36.0 Gy 31.5 Gy 26.0 Gy	33–40^a^	3–16^a^ 3–6 strictures	4–13^a^	0–1^a^
Rogers et al.^119^	284	6.5 Gy	6 (2 Implants)	39.0 Gy	1.5	0.6	0	0
Zamboglou et al.^17^	718	9.5 Gy 9.5 Gy 11.5 Gy	4 (1 Implant) 4 (2 Implants) 3 (3 Implants)	38.0 Gy 38.0 Gy 34.5 Gy	15.6 16.5 17.6	9.2 4.8 3.9	0 1.7 3.5	0.7 0 0
Ghilezan et al^126^	50	12.0 Gy 13.5 Gy	2 (1 Implant) 2 (1 Implant)	24.0 Gy 27.0 Gy	16	1	1	1
Barkati et al.^118^	79	10–11.5 Gy	3 (1 Implant)	30–34.5	2–6	2–4	0–3	0
Demanes et al.^69^	298	7.0 Gy 9.5 Gy	6 (2 Implants) 4 (1 Implant)	42.0 Gy 38.0 Gy	10	3	1	0
Mark et al.^117^	301	7.5 Gy	6 (2 Implants)	45.0 Gy	3.2	0	1.3	1
Martinez et al^120^	248	7.0 Gy 9.5 Gy	6 (2 Implants) 4 (1 Implant)	42.0 Gy 38.0 Gy	0.5–13 0.5 strictures	0.5–3 3 strictures	0–1	0–0.5
Ghadjar et al.^88^	36	9.5 Gy	4 (1 Implant)	38.0 Gy	25	11	6	0
Grills et al.^123^	65	9.5 Gy	4 (1 Implant)	38.0 Gy	3–15	0–3	0	0

Abbreviations: FACT‐P, Functional Assessment of Cancer Therapy—Prostate; IIEF, International Index of Erectile Function; IPSS, International Prostate Symptom score; QoL, quality of life; RTOG, Radiation Therapy Oncology Group.

^a^ RTOG toxicity scale (all other toxicity data according to Common Terminology Criteria for Adverse Events).

Contrary to the clinical data arising from definitive EBRT, the potential advantageous roles of temporary ADT for patients treated with HDR monotherapy remain an unresolved issue, fueling debate, as no convincing evidence exists,^25^, ^134^ with those debating against the addition of ADT suggesting that the increased intraprostatic dose suffices while those for ADT addition claiming that EBRT data should be equally adopted in this clinical scenario.

It needs to be stated that the excellent oncologic results of HDR BRT have prompted the implementation of stereotactic body radiotherapy (SBRT) for the treatment of localized prostate cancer employing extreme hypofractionation and utilizing continuous image guidance to automatically track, detect, and correct for intrafraction prostate movement.^135^, ^136^, ^137^, ^138^, ^139^ It seemingly combines “EBRT‐like” noninvasiveness with “HDR BRT‐like” biologic potency.^78^ However, a dosimetric analysis^140^ comparing virtual SBRT with actual HDR monotherapy plans from treated patients, indicated that HDR achieves significantly higher intraprostatic doses while, at the same time, provides similar urethral doses and comparatively lower maximum rectal doses. Notwithstanding this, SBRT, HDR, as well as LDR BRT have proven their efficacy, as safe for the management of localized prostate cancer. However, the validation of all the theoretical advantages as well as disadvantages of one modality over the other necessitate that randomized clinical trials are conducted, so that uncertainties concerning the clinical impact will be resolved. Adding to that, given the relatively restricted “surgical margin” associated with SBRT, it is clearly not recommended for more advanced disease presenting with extracapsular extension or seminal vesicle involvement.^136^, ^141^

In conclusion, HDR BRT as monotherapy proves to be an excellent modality for the management of low‐, intermediate‐, as well as carefully selected cases of high‐risk prostate cancer with long‐term follow‐up data justifying its safety and low side‐effect rate.

### 3.3 HDR monotherapy as salvage treatment

The optimal management of patients treated previously with definitive RT for clinically localized prostate cancer which are experiencing a biochemical recurrence (BCR) remains a challenging clinical issue,^142^ with various therapeutic managements put to the test, such as salvage radical prostatectomy (sRP), salvage high‐intensity‐focused US, and salvage EBRT (sEBRT) being clinically practiced.^143^, ^144^, ^145^, ^146^ Clinical evidence suggests that approximately 70% of patients with an increase in their PSA value will experience solely a local failure,^147^, ^148^, ^149^ devoid the variance in treatment‐related BCR definition.^150^, ^151^

Salvage HDR BRT (sHDR BRT) with or without ADT for clinically, histologically, and metabolically proven local recurrence after previous radical RT appears to be a safe, effective, and well‐tolerated therapeutic option which can be favorably compared with other nonradiotherapeutic local treatment modalities, in regard to disease control and toxicity rates.^149^, ^152^, ^154^ Considering that reports about local salvage modalities are in general scarce, only a few studies report the long‐term oncological outcomes following sHDR BRT. Even though all data arise from retrospective reports and are unfortunately relatively restricted in regard to patient sample size, with reported BC of the order of up to 77%, some of them have reached a 5‐year follow‐up. Table 6 lists the clinical outcomes of published studies reporting on sHDR BRT after definitive RT. In comparison to the primary BRT setting, an increase in adverse events is observed,^25^ although the toxicity rates are regarded as acceptable when compared to sRP and sEBRT. When compared with sRP series after previous definitive RT, symptomatic anastomotic strictures are reported in the range of 7%–41%, while GI toxicity focusing in rectal injury ranges in 0%–28%. At the same time, complete erectile dysfunction is of the order of 80%–100%, and complete urinary incontinence ranges from 21% to 90% of patients.^144^ Following sEBRT, late grade 3 GU adverse events of 7% to 18% have been reported.^162^, ^163^ With regard to LDR, no randomized trial has compared LDR and HDR neither in the primary nor in salvage treatment setting; however, nonrandomized evaluations have confirmed that both acute and late high‐grade toxicities are less frequent after primary HDR than LDR monotherapy.^123^ Similarly, late grade 3 GU and GI toxicity rates in the sLDR BRT literature range from 0% to 47% and 0%–20%, respectively.^157^, ^158^

**TABLE 6:** Oncological outcomes and late toxicity rates of salvage HDR protocols

Study	*n*	HDR protocol Gy/fraction	Fractions (implants)	Total dose (Gy)	Median f/u (months)	BC	Toxicity
Tharp et al.^91^	7	7.0 6.0 7.0 9.0	3 (1) 2 (2) 3 (2) 1 (2)	21.0 24.0 42.0 18.0	58	71.5%	28% Grade 3 GU No ≥ grade 3 GI
Lyszek et al.^92^	115	10.0	1 (3)	30.0	60	46% for GS ≤6	1.7% Urethral fistulas 1.7% Urinary incontinence 3.4% Bladder outlet obstruction
Pellizzon et al.^85^	17	8.5–9.0	4 (1)	34–36	47	70.5%	5.9% Late grade 4 urethral strictures 5.9% Late grade 3 GI
Jo et al.^155^	11	11.0	2 (1)	22.0	29	63%	No grade 3 GI/GU Low grade 2 GU
Chen et al.^148^	52	6.0	3 (2)	36.0	59.6	51.0% at 5 years	54% Late grade 2 GU 2% Late grade 3 GU 4% Late grade 2 GI 6% Late grade 3 sexual dysfunction
Oliai et al.^153^	22	6.0	3 (2)	36	45	95.5% at 2 years	18% Hematuria 32% Urethral strictures
Yamada et al.^156^	45	8.0	4 (1)	32.0	36	68.5% at 5 years	48% Late grade 2 GU 8.8% Late grade 3 GU 14% Late grade 2 GI
Kukieka et al.^157^	25	10.0	1 (3)	30.0	13	74% at 2 years	9% Late grade 2 nocturia 4.5% Late grade 2 obstruction 4.5% Late grade 2 frequency no grade 3 GU
Henriquez et al.^158^	19	Med. 5.25	1–4 (1–3)	17–39	48	77% at 5 years	21% Late grade 3 GU No late grade 4 GU 2% Late grade 3 GI
Hanna et al.^159^	28	Med. 6.0	Med. 6	Med. 36.0	83	DMFS 11% at 15 years	N. R.
Wojcieszek et al.^160^	83	10.0	1 (3)	30.0	41	76% at 3 years 67% at 5 years	39% Late grade 2 GU 13% Late grade 3 GU 6% Late grade 1 GI
Jiang et al.^161^	22	10.0	3 (3)	30.0	66	45% at 5 years	5% Late grade 2 GU 9% Late grade 3 GU 9% Late grade 2 GI
	139	6.0 8.0	6 (2) 4 (2)	36.0 32.0	61	45% for T3, 65% for T1‐2 at 5 years	11% Acute urinary obstruction 13% Urethral stricture

Abbreviations: BC, biochemical control; DMFS, distant metastases‐free survival; f/u, follow‐up; GI, gastrointestinal; GS, Gleason Score; GU, genitourinary; HDR, high‐dose‐rate brachytherapy; m, months; med, median; N.R., not reported.

Once again, the heterogeneity of clinically implemented protocols makes uniform recommendations concerning the optimal dose‐fractionation scheme for whole gland sHDR BRT trivial. However, the oncological results arising from single‐ or multiple‐implant regimes are considered consistent and reproducible, irrespective of the exploiting extreme hypofractionated or moderately hypofractionated treatment.

At the same time, sHDR BRT has been applied in the focal setting for the reirradiation of radiologically detectable recurrent disease.^166^, ^167^ Although it is clear that a significant dose reduction to OARs can be achieved by the implication of focal HDR BRT,^168^ further investigation is guaranteed to calculate the possible clinical impact both on morbidity and tumor control.

Currently, no consensus involving patient's eligibility for repeating a local therapy of organ‐confined recurrent prostate cancer exists, and the most suitable candidates have yet to be defined. Table 1 describes the selection criteria and contraindications. Nevertheless, the main rationale for HDR salvage treatment remains unchanged and is based solely on the presence of local disease in nonmetastatic patients, who are considered suitable candidates for radical therapy. The safe utilization of sHDR BRT either solely or as part of individualized treatment approach also for high‐risk patients is supported by an ever growing literature body.^148^, ^156^, ^157^, ^160^

## 4 CONCLUSION

HDR BRT is an excellent radio‐oncological modality for the management of prostate cancer granting an extraordinary low side‐effect rate. Valid mature follow‐up data support its safe and effective implementation in the treatment of prostate‐confined cancer regardless of risk group. However, further prospective and randomized studies are warranted to fully establish its role in clinically challenging prostate cancer cases.

## CONFLICT OF INTEREST

The authors have no conflicts of interest to declare.

## AUTHOR CONTRIBUTIONS

All authors had full access to the data in the study and take responsibility for the integrity of the data and the accuracy of the data analysis. *Conceptualization*, I.S., E.K., N.Z., K.F.; *Methodology*, I.S., E.K., N.Z., K.F.; *Investigation*, I.S., E.K., N.Z., K.F.; *Formal Analysis*, I.S., E.K., N.Z., K.F.; *Resources*, I.S., E.K., N.Z., K.F.; *Writing—Original Draft*, I.S., E.K., N.Z., K.F.; *Writing—Review & Editing*, I.S., E.K., N.Z., K.F.; *Visualization*, I.S., E.K.; *Supervision*, I.S., E.K., N.Z., K.F.; *Data Curation*, I.S., E.K., N.Z., K.F.; *Project Administration*, I.S., E.K., N.Z., K.F.; *Validation*, I.S., E.K., N.Z., K.F.

## ETHICAL STATEMENT

The authors are accountable for all aspects of the work in ensuring that questions related to the accuracy or integrity of any part of the work are appropriately investigated and resolved.

## Data Availability

Data sharing is not applicable to this article as no new data were created or analysed in this study.
